# Technical challenges related to implementation of a formula one real time data acquisition and analysis system in a paediatric intensive care unit

**DOI:** 10.1007/s10877-017-0047-6

**Published:** 2017-07-27

**Authors:** B. Rajeswari Matam, Heather Duncan

**Affiliations:** 1 Birmingham Children’s Hospital, NHSFT, Steelhouse Lane, Birmingham, B4 6NH UK; 2 Aston University, Aston Triangle, Birmingham, B4 7ET UK

**Keywords:** Critical care, Data recording system, New clinical data storage system, Technical challenges

## Abstract

Most existing, expert monitoring systems do not provide the real time continuous analysis of the monitored physiological data that is necessary to detect transient or combined vital sign indicators nor do they provide long term storage of the data for retrospective analyses. In this paper we examine the feasibility of implementing a long term data storage system which has the ability to incorporate real-time data analytics, the system design, report the main technical issues encountered, the solutions implemented and the statistics of the data recorded. McLaren Electronic Systems expertise used to continually monitor and analyse the data from F1 racing cars in real time was utilised to implement a similar real-time data recording platform system adapted with real time analytics to suit the requirements of the intensive care environment. We encountered many technical (hardware and software) implementation challenges. However there were many advantages of the system once it was operational. They include: (1) The ability to store the data for long periods of time enabling access to historical physiological data. (2) The ability to alter the time axis to contract or expand periods of interest. (3) The ability to store and review ECG morphology retrospectively. (4) Detailed post event (cardiac/respiratory arrest or other clinically significant deteriorations in patients) data can be reviewed clinically as opposed to trend data providing valuable clinical insight. Informed mortality and morbidity reviews can be conducted. (5) Storage of waveform data capture to use for algorithm development for adaptive early warning systems. Recording data from bed-side monitors in intensive care/wards is feasible. It is possible to set up real time data recording and long term storage systems. These systems in future can be improved with additional patient specific metrics which predict the status of a patient thus paving the way for real time predictive monitoring.

## Introduction

The National Patient Safety Association, National Institute for Health & Clinical Effectiveness and the Confidential Enquiry into Maternal and Childhood Deaths have all recommended the implementation of early warning systems to identify early clinical deterioration and initiate early treatment that can optimise recovery and improve clinical outcomes.

The need for extensive use of the information recorded to provide critical care to patients admitted in hospitals has received enormous support from healthcare professionals. Unlike other service providers such as insurance, banking, online retailers who have embraced information technology and the use of mathematical models to improve the efficiency of their service and use of their resources, healthcare providers have significantly lagged behind [1]. There is however growing acknowledgement that critical care is a complex information gathering and decision making process and the use of automated systems that could provide concise critical information would enable clinicians to possibly prevent adverse events [2–4].

Major healthcare information management systems were designed as basic data recording and viewing systems. The vital signs and physiological waveforms can only be viewed on bed-side monitors or a centralised computer but this data is not stored long term (duration of more than 4 days) and is not available for retrospective analysis. De Georgia et al. in their review of monitoring and data acquisition systems for patient care state that though patient monitoring systems were introduced in intensive care units in the 1960s, there has been very little advance made in the technology [5]. Physiological data is recorded using sensors attached to the patient and the data viewed on bed-side monitors. This data is analysed and scored by the bed-side nurses on A3 size colour coded paper charts at hourly intervals. The trends in the data recorded on the paper charts act as decision support mechanisms for the clinicians. Secondly most information systems in intensive care units do not have provisions to embed automated analytical techniques. Consequently large amounts of detailed trend information are lost resulting in a largely reactive rather than preventative approaches to healthcare management. Therefore in order to view the dynamical state changes of the patient’s physiology, systems capable of real time data analysis and long term storage with annotation facilities are needed.

In this paper we present the feasibility and implementation of a system which is able to capture, store and visualise data which could be used to create adaptive, patient-specific algorithms. We present the problems encountered, solutions applied and necessary evaluations that need to be undertaken in order to implement such systems. The clinical study outcomes will be reported separately.

## Current patient vital signs monitoring system

Vital physiological parameters are recorded from each patient in critical care with the help of sensors attached to the patient and viewed on Philips bedside monitors MP30 [6], a computational device which collects information from different biosensors as electrical currents and converts them into clinically relevant data such as waveform data (for example ECG, PPG) and vital signs (for example heart rate, respiration rate, oxygen saturation). The data from the bedside monitors is transferred to a Philips IntelliVue Information Centre (IIC) iX, central station where the data from all the beds can be viewed simultaneously.

The limitations of the current system include

- An inability to store data for longer than 96 h.
- The trends in the data (a 1 min minimum period between each data value) can be printed as waveforms but the numerical values cannot be exported.
- An inability to embed code developed externally that is capable of real time analysis.

## Context

This study was part of Young Lives project conducted in Birmingham Children’s Hospital National Health Service Foundation Trust (BCH NHSFT) PIC between May 2011 and April 2014. As there are limited real time platforms for capturing continuous physiologic data in critical care, we took the unique and novel approach of partnering with McLaren Electronic Systems, Woking, UK [7] to translate their expertise used to continually monitor and analyse the data from F1 racing cars in real time into a similar system adapted to the needs of the intensive care environment.

## Ethics and consent

Ethical approval was awarded for ‘opt out’ consent by the Local Research Ethics Committee (LREC, West Midlands—South Birmingham Research Ethics Committee) [8], LREC reference number 11/H1202/13. Parents/carers of the patients were informed about the study by research nurses and requested for consent. As the data was recorded in real time from the time of admission to PICU, the data of patients who opted out was deleted from the database and was not utilised in the study.

## Software

The project involved the installation of real-time data recording software, ATLAS from McLaren Electronics Systems [7] on a virtual data server on the BCH computer network. The parameter data recorded by the bedside monitors, Philips MP30 was transferred to the Server in real time. The data is stored in Microsoft SQL database through the SQL Race. SQL Race is the interface software that transfers data to the database from external systems, includes pointers to the data and transfers data (segments requested by the user) to a Graphical User Interface (GUI) that can be used to view the data. Additional data analytics software implementing automated early warning scores, machine learning and nonlinear signal processing algorithms using MATLAB 8.5 [9] was provided as an interface between the data collection system and the clinician interface to the data system by Aston University.

## Database recordings

**Fig. 1:**
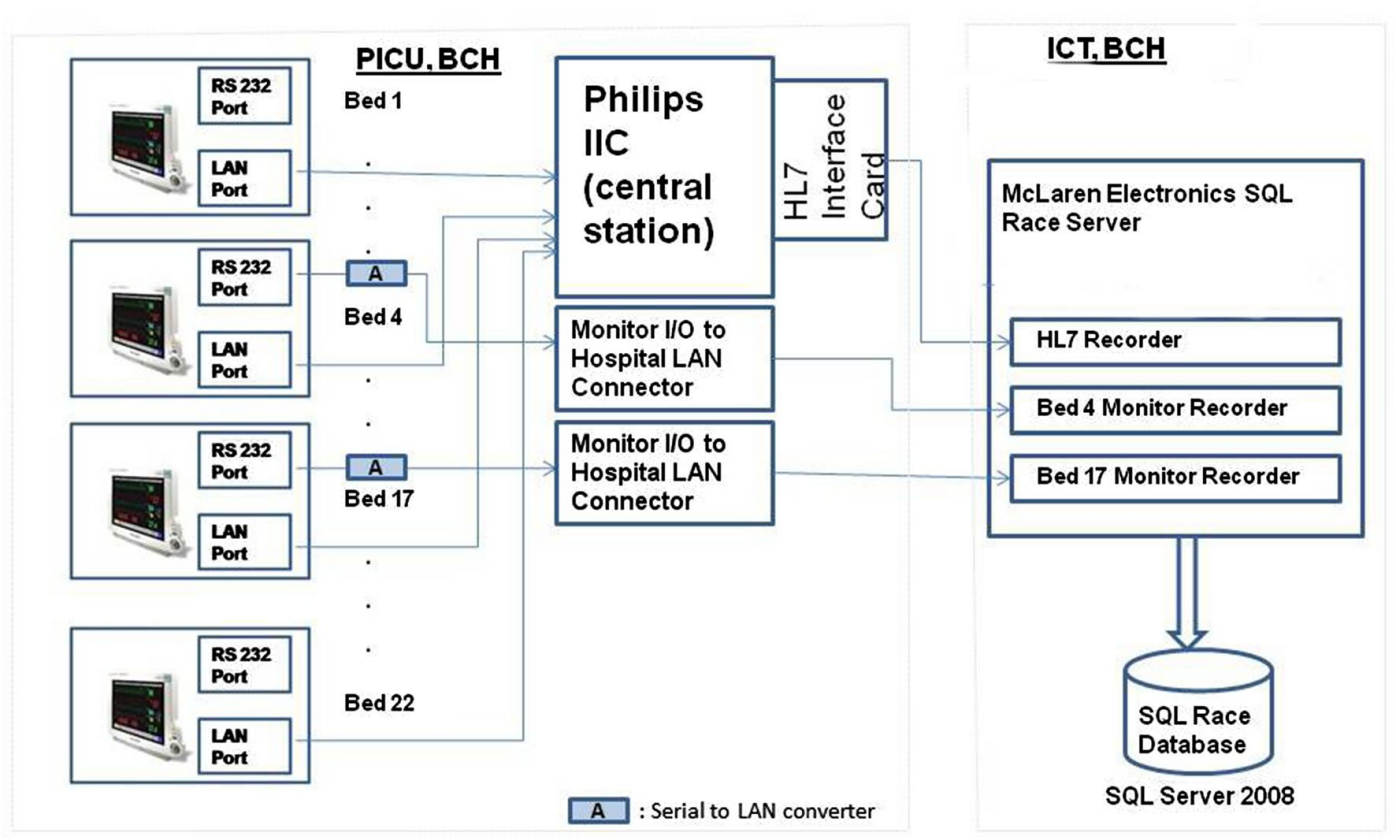
Overview of system set up in PICU, BCH

The database is a collection of physiological data from three different sub systems as explained below and shown in Fig. 1.

### Data recorded in the IIC

The IIC contains an electronic record of each patient in the PIC. However the data exported from the IIC is a coarse representation of the physiological data representing trend information at a maximum frequency of 0.016 Hz limiting detailed exploration by clinicians.

In comparison the SQL-Race application records the data on the IIC through a HL7 [10] interface card in real time at a frequency of 0.2 Hz. The advantages of this system include a recording of data which is fine grained, contains in-depth information of the physiological data of a patient and allows clinicians to view and review cases at any time.

**Fig. 2:**
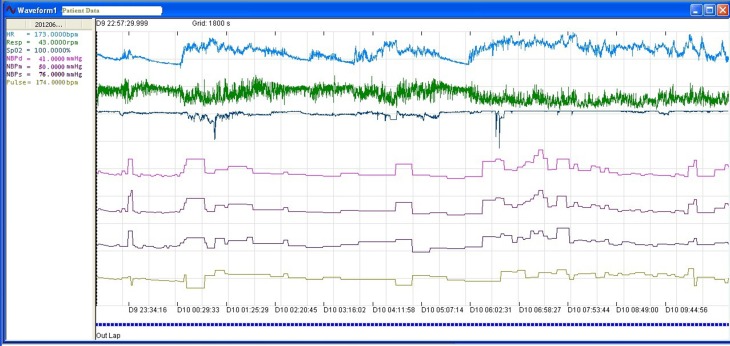
Physiological data as viewed on ATLAS

Figure 2 shows the physiological data of a patient as viewed on the ATLAS. The duration between two grid lines is equal to 30 min. The time at which each data point was recorded was represented as the day of admission followed by a 24 h clock. For example if the data was recorded on the ninth day of the patient’s stay in PICU at 23 h, 34 min and 16 s, the time stamp was ‘D9 23:34:16’. The data on the ATLAS could be zoomed in to view fine details or zoomed out to view the trends in the data over multiple days or the entire length of stay of the patient. The signals were separated along the y-axis and represented using different colours to provide for clear visualisation.

### Data from the bedside monitors

One of the limitations of the data recorded from the IIC is the loss of waveform data. The shape of the individual ECG heart beats and the photo plethysmograph waveforms provides vital information to the clinician. The waveform data was streamed from two of the beds using a parallel network connection bypassing the IIC. The bedside monitors contain two Input/output (I/O) ports. One I/O port is a RS232 port and the other a serial I/O bus. Data from the bed-side monitors is transferred to the IIC via wired connection through the RS232 port. Data from the IIC can further be transferred to external servers using HL7 messaging service however only parametric data (for example heart rate, respiration rate) can be transferred. The waveform data such as PPG and ECG cannot be transferred. Therefore a serial device server was utilised to connect the serial I/O port to the PICU LAN and the waveform data was transferred to the SQL Race Server.

In summary, there were two parallel data feeds to the SQL Race Server from the PICU. One included the vital signs recorded at 5 s intervals transferred through the HL7 port from the IIC wirelessly and the other waveform data recorded at 100 Hz from the bedside monitors directly via the LAN. Comparison of the two data systems was not conducted as it was outside the scope of the project.

## Advantages of this system

- The ability to alter the time axis to contract or expand periods of interest.
- The ability to store and review ECG morphology retrospectively.
- Detailed post event (cardiac/respiratory arrest or other clinically significant deteriorations in patients) data can be reviewed clinically as opposed to trend data providing valuable clinical insight. Informed mortality and morbidity reviews can be conducted.
- Storage of high rate data capture to use for algorithm development for adaptive early warning systems.
- Export the data and conduct offline analysis mathematically to develop alternate visualization techniques.

**Fig. 3:**
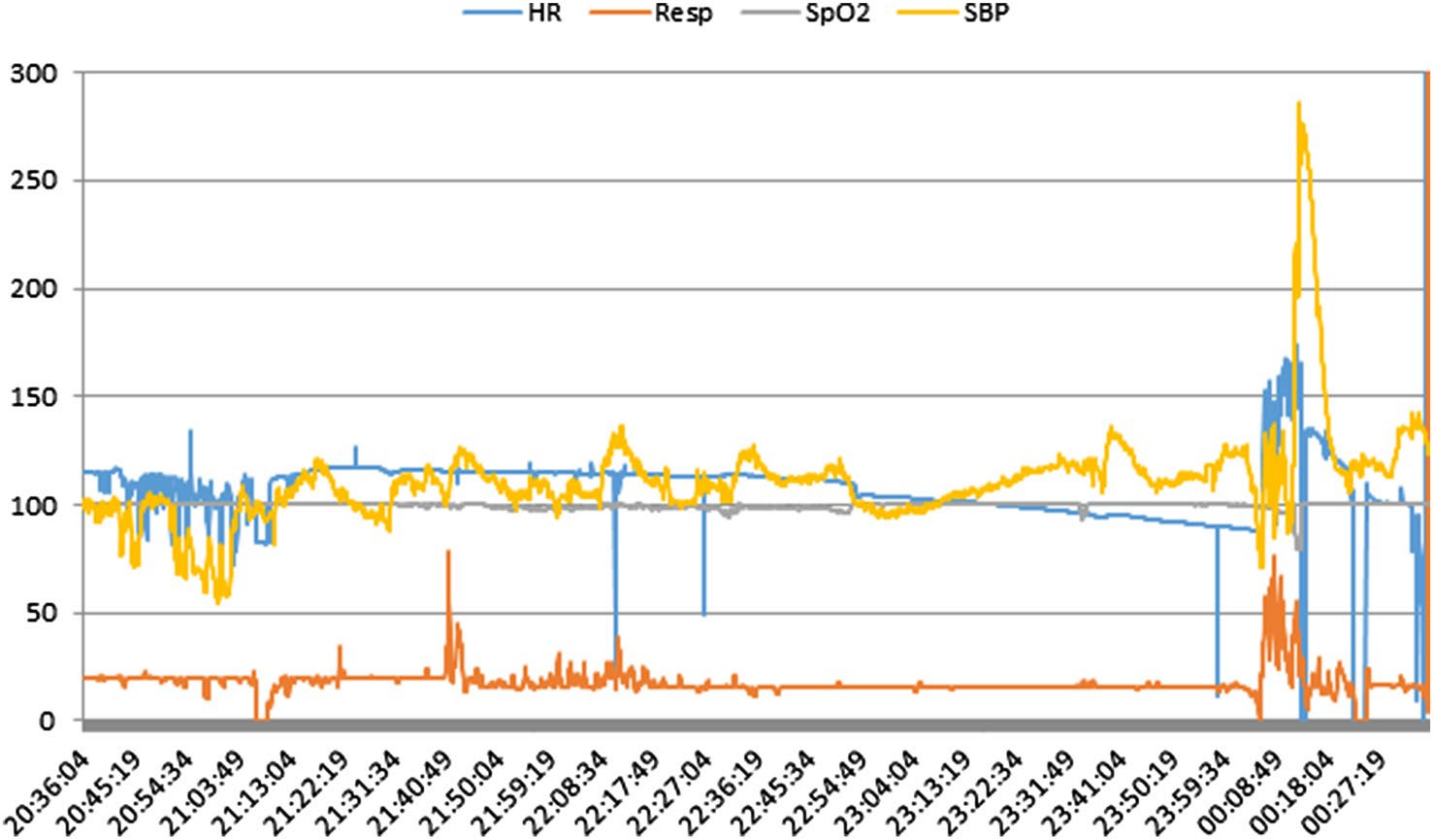
Visualisation of vital signs of a patient who experienced a cardiac arrest

Figure 3 shows an example of how the vital signs of a patient, who experienced a cardiac arrest, can be exported into different formats and plotted for post-incidental analysis by clinicians. This cardiac arrest was listed as unpredictable because the bed-side monitors display the latest 20 s of waveform data (ECG, PPG) and the current values of the vital signs. From the figure it can be seen that there are significant deteriorating trends in the vital signs leading up to the cardiac arrest. Real time visualisation of these trends if enabled could provide advance notification of deterioration to the attending clinicians. This could result in time critical intervention and stabilisation of the patient leading to possible better outcomes for the patient.

## Issues with system implementation and maintenance

The work processes in an established PIC are complex and fluid and, the information systems in some cases outdated. Introducing any new technology will entail disrupting routine clinical care and therefore requires extensive planning and a multi-department level integration. One of the main reasons for the failure of expert systems in clinical environments includes poor execution plan of the expert systems [5].

The system implemented in this project consisted of two individual PCs, one representing the Philips central station (with data displayed on a monitor and keyboard to input data) and the other containing the ATLAS software (a CPU with no input/output devices connected).

As this was the first of its kind system to be implemented, dependence on the vendor’s knowledge and experience in the implementation of the system was high however the vendor’s lack of knowledge of the organisational structure of a PICU/hospital proved to be a major challenge. The system was initiated and tested for connectivity for a period of 6 weeks before patients were consented for the use of their data. The data of patients recorded during this period was deleted. Various functionalities of the system including installation of the ATLAS, clinical data authentication, protocol for automated anonymisation of the data files on the server, modifying the export formats of the data files to suit clinical requirements (which were different from those used during F1) were tested during this period. This testing did not however flag up the various issues encountered during the project. This could be because the system was not running continuously but was switched on and off during this period.

**Fig. 4:**
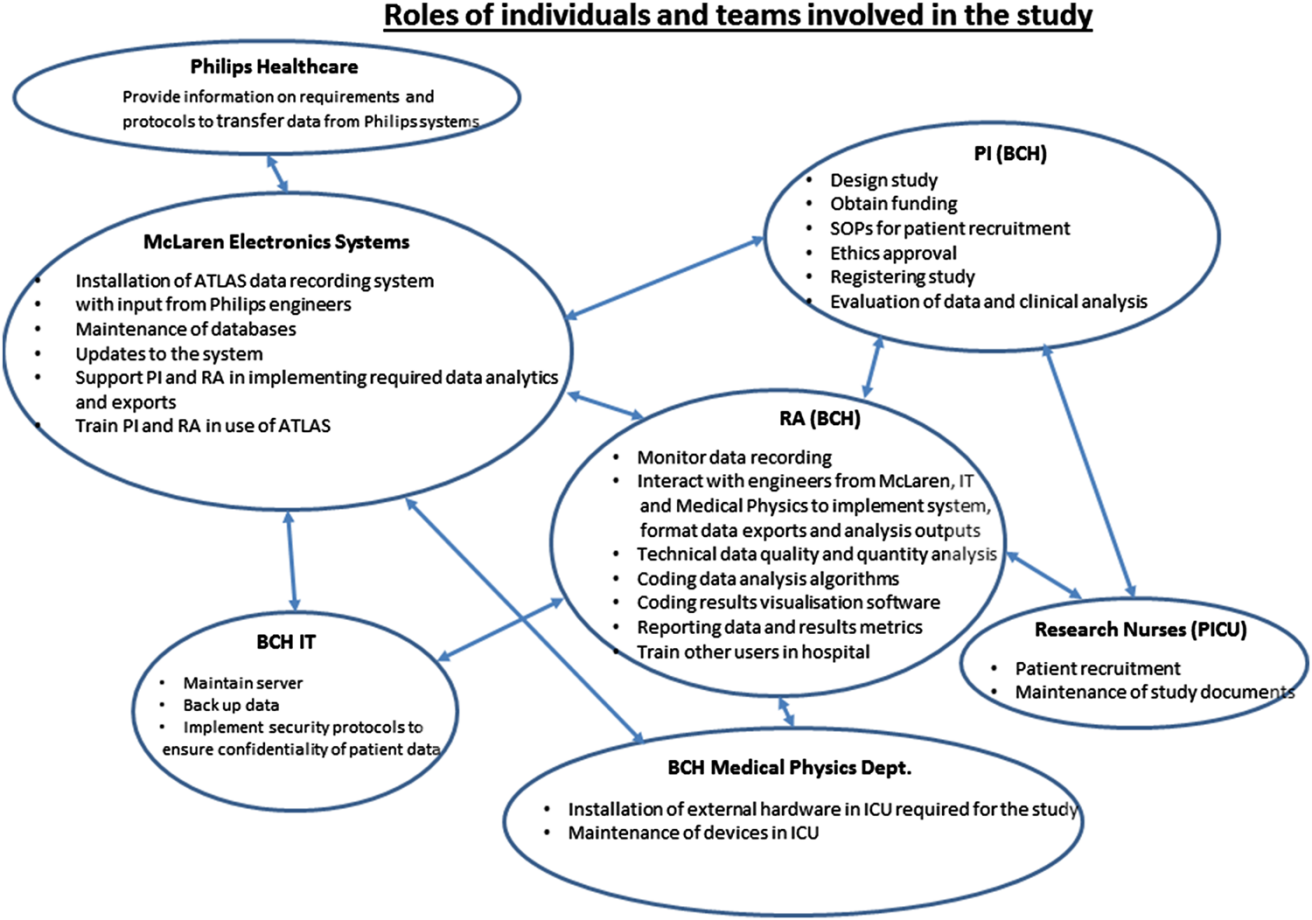
Roles and responsibilities of the teams in the study

Figure 4 shows the roles and responsibilities of the various individuals and teams involved in the study. The devices and information systems in the PICU were managed by different organisations (external) and departments within the hospital. Sensors were bought from different manufacturers. Philips Healthcare provided the bed-side monitors which recorded the patient’s vital signs and physiological data and the central server for collation of data from all bed-side monitors. Medical devices were tested and serviced by the medical physics department of the hospital; bed-side monitors and the central server were installed and serviced by Philips; databases containing patient information were maintained by the hospital Data Informatics department whilst the physical computer systems including computer networks both wired and wireless were maintained by the hospital IT department.

The first step at initiation of the project was interfacing with the Philips central station. This required clarification of the structure and roles of individuals within the medical physics, IT and data informatics departments of the hospital. A key delay was caused when the communication between the two systems, the Philips central station and the ATLAS was to be initiated. The communication port address of the central station was known only to Philips while the IP address of the central station was known only to the server specialists in IT and not the front line support of the IT department. Connectivity was achieved after multiple communications.

Two major causes for the data loss when the system was fully operational are discussed in detail. The SQL Race database application was built using the Microsoft SQL Server File Stream technology. This technology did not support too many files (limit of 300,000) in the file stream folder. The SQL Race application was built to record data from a single car for one hour from more than 100 sensors however this application was used to record data from 28 beds continuously 24 × 7. The size of the data recorded in a 24 h period ranged between 20 and 50 MB, this data was analysed continuously using specialist data analysis software and the results of the analysis were also stored with the data. Database overload occurred every 3–4 weeks leading to server crash. Though a permanent solution could not be implemented during the life of the project, temporary measures such as creating new databases after every crash were implemented.

PICU, BCH assigns a unique numeric ID to every patient admitted for administrative purposes. The numeric ID is of 8 digits length starting with the year followed by a four digit number. For example the first patient admitted to PICU in the year 2017 is labelled as 20170001. This number is different to the patient’s NHS and Hospital number. Patients transferred to the PICU need immediate and urgent care. This resulted in the admission process and the patient being allotted a PICU number being carried out a few hours after the actual admission. In the case of patients who were admitted after 5 pm, there would sometimes be a delay of 24 h from admission for the patient to be allotted a PICU number. As the database was maintained by external industry partners, LREC permission for data recording included the anonymisation of patient data to ensure patient privacy and confidentiality. It was agreed to use the PICU number to tag the patient data as knowledge of the PICU number does not provide additional details such as name, age, address or any other details. The probability of identifying the patient with the help of the PICU number is extremely small as the records that relate the PICU number to the NHS number were not accessible to non-PICU staff. Secondly the industry partners did not have permission to upload or download any file to/from the project server; permission to modify the files on the server was restricted to the IT staff in BCH. The PICU number was entered on the bed-side monitor once allocated and the data session would be recorded with this identifier. The data of a patient from the time of admission to PICU until they were allotted a PICU number was therefore recorded as an unknown data session. It is also common practise to move patients across beds based on emerging clinical needs such as access needed by multiple clinical teams. The sessions had to be renamed using identifiers such as bed numbers and other patient details but in some cases this was not possible as the patient had moved beds before the PICU number was assigned. Also the sheer volume of admissions and discharges did not permit the identification of all the sessions. Only the data sessions of patients who had experienced a cardiac or respiratory arrest were re-identified. Almost 20% of the patient data recorded was therefore recorded as anonymous.

There were other standard maintenance protocols which caused disruption to the data collection phase. The cause of some of the problems could not be understood immediately. They are as shown in Table 1.

**Table 1:** Issues encountered in the implementation of the system and solutions applied

	Issue	Cause	Solution	Team involved in resolving the issue	Time taken to resolve the issue (duration)
1	Switching off PC used to record data from the Philips central station	Porters, cleaners and nursing staff intermittently disconnected the PC containing the ATLAS software as its significance was not known	Relocate the PC to a secure office	Medical Physics department	45 days from start of project with the issues occurring three times
2	Switching off of PC on weekends	Hospital back up power/ generator tests	Connect the PC with ATLAS software to an uninterrupted power supply	Medical Physics department	Solution provided after the system was shut down four times on four alternate weekends (2 months) following generator testing
3	System shut down once a month	Implementation of software updates on all the computer systems turning off the systems after the updates	Security updates were disabled. Access to system limited to core research team	BCH IT	3 months from start of study
4	PC with ATLAS software crashed and could not be recovered	The PC could not cope with the high load of operations executed continuously 24 h a day	The system was re-implemented on a dedicated server (100 GB) at extra cost	McLaren engineers, BCH IT	1 month
5	Transfer physiological signals such as ECG, PPG recorded at 127 Hz from Philips central station	Philips IIC does not have output ports for the transfer of this data	Serial to LAN convertors bought from external vendors connected between RS232 port of bed-side monitors and PICU LAN	Medical physics, McLaren engineers and RA	6 months
6	ATLAS system disconnected from Philips central station. No data was recorded for 3 months	The PICU was upgraded from a 22 bed unit to a 30 bed unit. This required major changes to the Philips IIC which was executed by Philips engineers in collaboration with the unit nurse in-charge. The project team was not included in the decision making process nor the lead nurse had detailed knowledge of the project. The PC used for the IIC was replaced by a different PC effectively cutting off the data transfer	Re-initialisation of the project. Three months of data was lost over this period	BCH IT, McLaren engineers, RA and Philips engineers	3 months
7	Shut down of server at night	Database back up process disconnected the SQL Race application from the Philips IIC	Changes to the SQL Race application enabling auto re-connection a minimum of 20 times every time it was disconnected	RA, McLaren engineers	2 weeks
8	Server crash every 3 weeks	SQL Race database overload	Creation of new databases after every crash	RA, McLaren engineers	3–4 h every 3 weeks
9	Data loss	Admission process not suitable for technical purposes	Data re-identified retrospectively based on information such as admission time and bed number	RA	An hour every couple of weeks
10	Multiple identification numbers for data of same patient with multiple admissions to PICU	The real time system could not tag the data of the same patient if admitted multiple times to the PICU as belonging to the same patient as the data was identified using the PICU number which is allotted per admission	Data re-identified retrospectively based on confidential hospital numbers for patient which is unique and allotted only once for each patient	RA	An hour every couple of weeks

## What we have learnt

With the experience gained during the implementation of our clinical information system, we have learnt that implementing new clinical systems needs a multidisciplinary project team consisting of clinical, IT (server specialists), medical physics, software engineers and data analysts. IT staff, especially those with expertise in computer networks and servers, should be included in the planning, design, selection, assessment and revamping of the computing systems. Different aspects of planning included:

Practical:

- It is vital to understand the data flow processes, the systems currently in use and the IT networks both wired and wireless for usage and efficiency.
- Label all equipment clearly including signs on power points to avoid accidental unplugging.
- Signpost clinicians to contact the individual researchers responsible for the system.
- Establish uninterruptable power sources and protocols for generator testing and power outage.
- Establish routines for pre-warnings before and system integrity checks after major software upgrades.
- Secure data storage. A secure virtual server maintained by the IT personnel will ensure the running of the system continuously with daily back-up similar to routines for clinical data as a precautionary measure against an unseen failure.
- Data collection should be reviewed weekly and actively managed up to aim for 100%.
- Develop a system to ensure all data is correctly labeled for each patient. As a requirement of research ethics our data was anonymously labeled (and sometimes mislabeled).
- Usability testing for visualizations should be undertaken to enhance utility.

Risk assessment:

- Workflow processes and procedures must be analyzed for risks by all the staff including those who will be involved remotely such as external vendors. Risk mitigation needs to be prospective and retrospective. Despite this unexpected events need a quick response to reduce data loss.
- Monitor and anticipate memory capacity issues as a routine. Memory leaks and excessive data storage are common even if server capacity is estimated and established at the start of the project.

Within team communication and dissemination:

- The aims change throughout the lifecycle of the project.
- It is imperative to communicate and confer regularly with all the teams to encourage joined up and creative solutions to problems.
- Conduct project dissemination workshops and advertise the project to all the staff within the establishment to ensure early reporting of any probable issues.

## Statistics of data recorded

Data was collected on the number of patients screened and consented. Ethical approval was awarded for ‘opt out’ consent and parents were approached by research or bedside nurses and informed about the study.

Table 2 provides information about the number of patients admitted and recruited to the study. The ‘Opt out’ consent yielded a high number of recruits. The 10% of cases not recruited were from high turnover patients and non-English speaking parents which led to the inability to document that families had been given information about opting out. Only 8 families declined to participate because they did not want to participate in a research activity.

**Table 2:** Demographics of patients and number of hours of data recorded

Age in years (Total number admitted to PICU)	Recruited to study	Number of hours of physiological data recorded
Birth to ≤1 (1994)	1768 (89%)	148759.8
>1 to ≤18 (1844)	1763 (96%)	87204.1

**Fig. 5:**
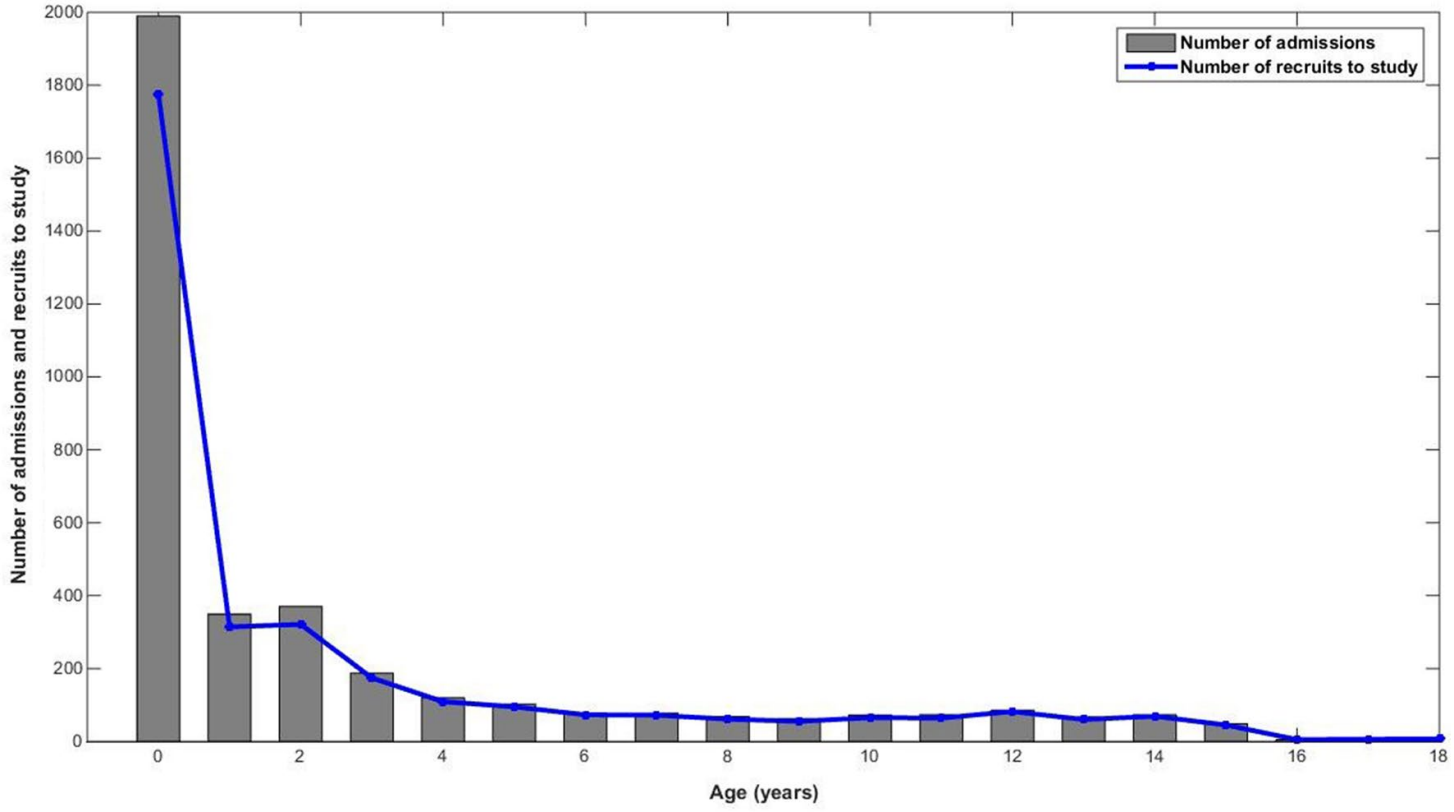
Demographics of admission numbers and recruitment numbers across different ages

Figure 5 shows the number of admissions and the number of recruits per year of age. It can be seen that the recruitment rate to the study was not dependent on age and is nearly equal across all the different ages. We compared the patterns of recruitment for the different age groups for the cohort of patients admitted to the PICU using the χ^2^ statistic and found that the recruitment rate was not dependent on the age of the patient (p = 0.65).

**Fig. 6:**
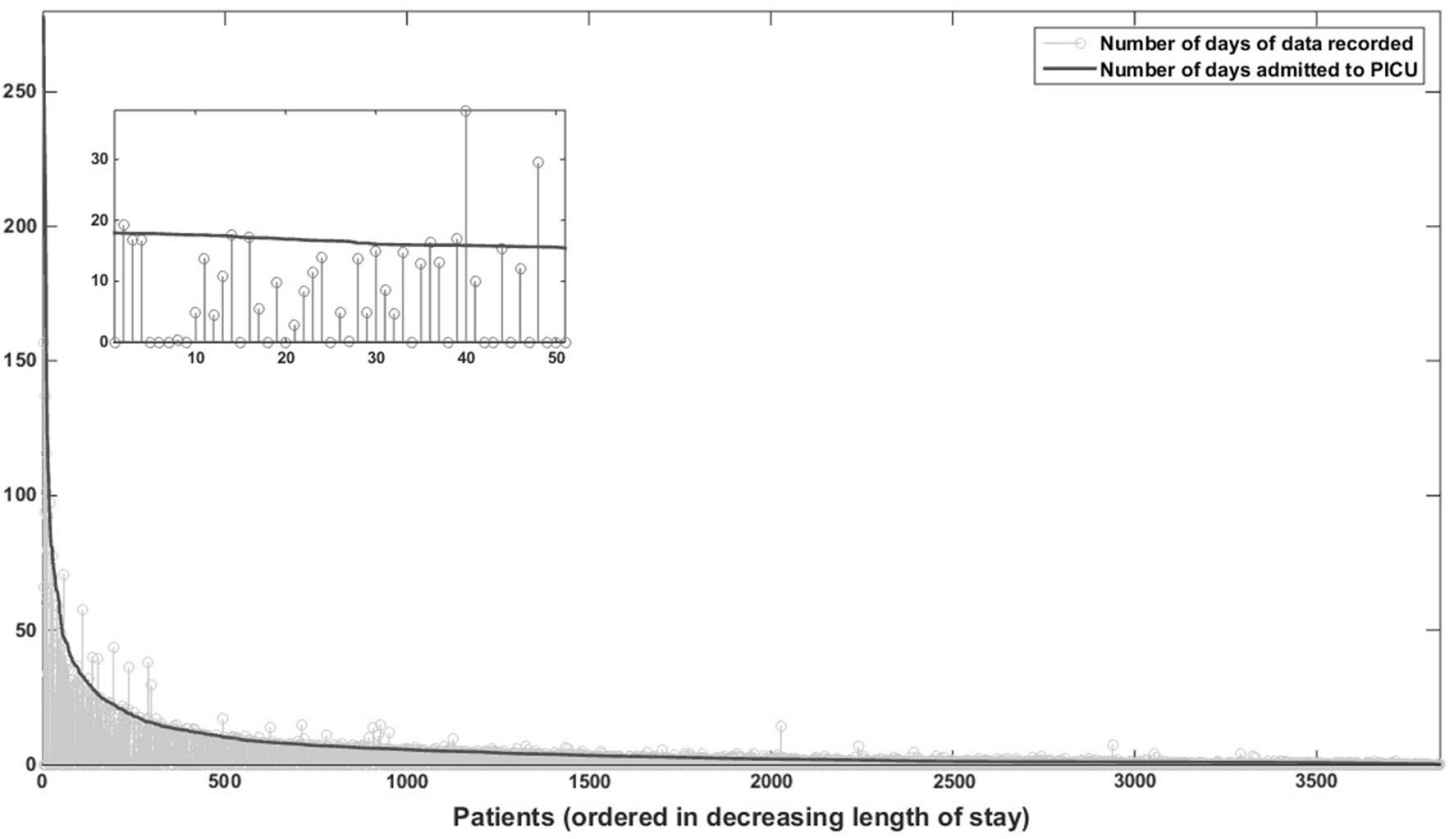
Length of stay (days, *black dot*) of each patient recruited to the study and the physiological data recorded (days, *grey line*)

Figure 6 shows the length of stay (days, black dot) of each patient recruited to the study and the physiological data recorded for that patient in terms of days in grey in decreasing order of length of stay. Due to the large number of recruits to the study, a zoomed in version of the plot for a random group of patients whose length of stay was less than 20 days has been included. It can be seen that some of the patients’ data was lost. While the length of the data recorded is less compared to the length of the stay for most of the patients, some of the patients had more data recorded compared to the length of the stay. This anomaly was due to the incorrect admission and discharge dates and times recorded and as the consent was opt-out, the patients’ data was recorded prior to the consent and hence this error could not identified or corrected.

Table 3 shows the number of recorded and unidentified data records and the number of high rate data records in terms of sessions. Each session represents the data for a patient for approximately 12 continuous hours. The total size of the data recorded over the 3 years of the project duration is ~29 GB. This included only the parametric data sampled at 0.2 Hz from 22 beds for 3 years, parametric data from eight beds for one year and waveform data sampled at 125 Hz from two beds for 2.5 years.

**Table 3:** List of patients admitted, recruited to the study and data sessions recorded

1	Number of patients		Admitted to PICU: 3838	Screened and recruited: 3531 (92%)		
2	Data sessions	HL7	Recorded: 42,617	Identified: 36,998 Unidentified: 1215 (2.8%) Mislabelled: 4404 (10.33%)	Size of total recorded data ~23 GB	Total number of hours of data recorded: ~511,404
		High rate sensor data	Recorded: 1516	Identified: 1261 Unidentified: 255 (20%)	Size of total recorded data ~6 GB	Total number of hours of data recorded: ~18,192

## Discussion

We have demonstrated that a software platform providing the ability to perform real-time analytics based on commonly measured physiological data, can be provided within a hospital using software that is routinely used in other service industries. Building a ‘research’ system in a clinical environment has significant challenges and we have outlined the major technical and process challenges.

Only 0.2% of families elected to ‘Opt out’ which confirms that ‘Opt out’ consent is an appropriate way to collect large datasets. Families expressed the view that it was essential that we collected and learnt from vital signs already being measured. This has implications for future ‘Big data’ studies and should be explicitly explored to identify families’ preferences and attitudes to risks of collecting and analysing data.

Data loss in new systems is the major problem. The confidentiality requirements of this study led to a further 20% of sessions being unlabelled. Our future studies all include this research data as part of the patient clinical record to reduce loss due to data loss. De-identification is conducted later during the analysis phase to reduce this source of data loss. The remaining sources of data loss were technical systems failures, system power loss, memory leaks and unanticipated upgrades. A study of the routine clinical and housekeeping maintenance procedures in the PICU and the hospital in general and, adapting the system used in a domain unrelated to healthcare with the user requirements would have reduced the issues encountered in our project.

The main problem is the lack of guidelines from manufacturers of physiological data monitoring devices on the output ports and communication protocols with external storage systems [4]. Physiological signals such as the ECG and PPG could not be recorded through the HL7 port. Documentation on recording, interpreting the numerical values retrieved and storing these signals was not available. Engineers from the Medical Physics and McLaren team and the RA resolved the issue through brainstorming, self-study and trial and error. Different manufacturers design the same devices with no common standard for intra-device communication. A comparison of various physiological databases has been presented in [11]. The data recorded in each database varies from the type of data recorded to the duration of the data recorded. We recommend that healthcare systems providers should provide technical details on adapting and integrating the systems with external data analysis software for research purposes.

In the system implemented in our study the cost of servers, licences, IT support, engineers’ salaries was nearly £500k/year. The system requires major changes in terms of database structure and analytical software to suit the requirements of healthcare. Follow on funding from the Wellcome Trust is developing the system on two cardiac wards.

Existing paediatric scoring systems mainly tested in ward based patients, which are expert-derived, show reduced mortality, cardiac and respiratory arrest rates [12]. Early Warning Systems are deployed exclusively outside of intensive care and there are few reports of systems deployed in high dependency areas [13–15]. The data recorded by the system described in this paper was utilised to retrospectively analyse the data. Future work includes extending and implementing a real time automated scoring system and analysis system of the mathematical models with further funding. The new system would enable (a) direct visual trend information to enable clinicians in enhanced, streamlined, timely, patient-focused clinical decision-making and (b) alternate physiological data visualization techniques capable of providing personalized predictive trends capable of identifying possible deterioration in the health status of the patient.

## Conclusion

Life-threatening events in children are frequently preceded by early warning signs. However, these are frequently missed or not acted upon. This is because most current monitoring systems do not provide the accuracy required in paediatrics, nor can they provide the continuous analysis of the monitored physiological data that is necessary to detect transient or combined vital sign indicators. Recording and analysing the physiological data such as ECG, PPG, heart rate and other vital parameters in real time from bed-side monitors in intensive care/wards is feasible. There are significant challenges however during the installation and maintenance of different Health Information Systems. Due to various issues, nearly 20% of the recorded data was unidentified and, around 15% of the data was lost due to software, hardware and other issues. There is limited literature related to the source and magnitude of these challenges which we have tried to address in this paper. Better data identification and reduced technical failures are required to ensure the system can be used reliably for early warning. It is possible to set up real time analysis systems which provide additional patient specific metrics which predict the status of a patient thus paving the way for real time predictive monitoring. A further report will detail the predictive algorithms developed and compare them with clinical predictions of early warning.
